# Metabolic effects of high-dose glucocorticoid following out-of-hospital cardiac arrest

**DOI:** 10.1186/s40635-025-00754-8

**Published:** 2025-04-26

**Authors:** Rasmus Paulin Beske, Laust Emil Roelsgaard Obling, Martin Abild Stengaard Meyer, Jacob Eifer Møller, Jesper Kjaergaard, Pär Ingemar Johansson, Christian Hassager

**Affiliations:** 1 Department of Cardiology, Rigshospitalet, Copenhagen University Hospital, Blegdamsvej 9, 2100 Copenhagen, Denmark; 2 Center for Endotheliomics, CAG, Department of Clinical Immunology, Copenhagen University Hospital – Rigshospitalet, Copenhagen, Denmark; 3 Department of Cardiology, Odense University Hospital, Odense, Denmark; 4 Department of Clinical Medicine, University of Copenhagen, Copenhagen, Denmark

**Keywords:** Cardiac arrest, Glucocorticoid, Methylprednisolone, Metabolomics, Inflammation

## Abstract

**Background and aim:**

Patients resuscitated after out-of-hospital cardiac arrest (OHCA) face high morbidity and mortality rates, primarily due to ischemia–reperfusion injury, a complex metabolic disorder that triggers a significant systemic inflammatory response. Glucocorticoids mitigate inflammation but also impact the cells beyond the immune response. This study aims to identify glucocorticoid effects on plasma metabolites.

**Methods:**

This explorative sub-study is part of a two-center, blinded, randomized controlled trial (NCT04624776) examining the effects of high-dose glucocorticoid on comatose patients resuscitated from OHCA of presumed cardiac origin. Following resuscitation, patients received 250 mg of methylprednisolone or a placebo in the prehospital setting. Blood samples were collected upon hospital admission and 48 h later. Sixty metabolites were quantified in the plasma using mass spectrometry and compared between groups.

**Results:**

In the modified intention-to-treat population, 68 patients received methylprednisolone, and 69 received placebo [median age was 66 years (IQR: 56–74) and 83% were men]. Blood samples were available for 130 patients, 121 (88%) at admission and 117 patients (94% of patients alive) after 48 h. Although a nominal difference was observed at admission, no significant metabolic effects were found after correcting for multiple testing. After 48 h, the placebo group had 83.4% (95% CI 16.9–187.6%) higher prostaglandin E2 and higher levels of linolenic acid and arachidonic acid. The methylprednisolone group had higher levels of tryptophan (47.6%; 95% CI 27.9–70.2%), arginine, and propionylcarnitine (C3).

**Conclusions:**

In this exploratory study, early administration of 250 mg of methylprednisolone after resuscitation appeared to drive sustained metabolic effects over 48 h. Specifically, methylprednisolone led to reductions in ω-6 fatty acids and increases in several amino acids, with a notable rise in tryptophan.

**Supplementary Information:**

The online version contains supplementary material available at 10.1186/s40635-025-00754-8.

## Introduction

Unexpected out-of-hospital cardiac arrest (OHCA) is a leading cause of death worldwide [1]. Despite advancements in resuscitation techniques, the mortality rate remains high due to anoxic brain injury and circulatory collapse [2]. In the immediate aftermath of cardiac arrest, patients frequently experience mitochondrial injury and metabolic disturbances resulting from ischemic reperfusion injury that triggers a systemic inflammatory response, key features of post-cardiac arrest syndrome (PCAS) [2–4]. These factors significantly contribute to decreased survival rates.

The *prehospital high-dose methylprednisolone in resuscitated out-of-hospital cardiac arrest patients* (STEROHCA) trial was designed to investigate the potential benefits of early glucocorticoid intervention in OHCA patients [5]. The STEROHCA trial aimed to address the systemic inflammatory response through early administration of methylprednisolone, hypothesizing that it could mitigate organ and cellular injury by modulating the inflammatory response. The trial demonstrated that methylprednisolone effectively reduced levels of interleukin-6 (IL-6), indicative of a dampened inflammatory response [5]. A secondary analysis of the STEROHCA trial found that patients treated with methylprednisolone had lower requirements for pharmacological circulatory support, possibly due to the shock-reversal effects of glucocorticoids [6, 7].

This sub-study focuses on the metabolomic analysis of patients randomized to receive either methylprednisolone or placebo. Metabolomics, the comprehensive study of small molecule metabolites within biological systems, offers a powerful approach to uncovering the biochemical alterations associated with disease states and therapeutic interventions [8]. By identifying specific metabolites that differentiate between treatment groups, this study aims to enhance our understanding of the metabolic pathways affected by glucocorticoids.

The primary objectives of this study are to assess the impact of prehospital methylprednisolone administration on metabolite concentrations at 48 h and to explore the associations between metabolite levels, hemodynamic profiles, and clinical outcomes.

## Methods

### Design, study population, and study intervention

This explorative sub-study is part of the STERoid as anti-inflammatory and neuroprotective agent following resuscitated OHCA (The STEROHCA trial). The design of this trial has been comprehensively described [5, 9] and is registered on https://clinicaltrials.gov with the identifier NCT04624776. In brief, the STEROHCA trial was an investigator-initiated, randomized, placebo-controlled, blinded phase II trial conducted in the Capital Region of Denmark from October 10, 2020, to July 15, 2022.

The trial was carried out, in collaboration with the Emergency Medical Services, at two cardiac arrest centers in Denmark, serving the Capital Region of Denmark, which has a population of 1.9 million inhabitants.

Eligible OHCA patients were screened by the prehospital physician in the critical care unit responding to the OHCA incident. Patients had to be 18 years or older, have experienced an OHCA presumed to be of cardiac origin, remained comatose (GCS < 9) after the return of spontaneous circulation (ROSC), and maintained ROSC for at least 5 min. Key exclusion criteria were as follows: asystole as the first monitored rhythm, women of childbearing potential, a known pre-arrest modified Rankin Scale (mRS) score of 4 to 5, a body temperature below 30 °C at the time of randomization, or a time to ROSC exceeding 30 min. For the complete list, refer to reference [5].

If deemed eligible for inclusion, patients were randomized to receive either a bolus injection of 250 mg of methylprednisolone intravenously (two 125 mg/2 mL doses) or a placebo consisting of 4 mL isotonic NaCl, with both administered over a 5-min period. The intervention was carried out as soon as feasible after resuscitation, with a minimum delay of 5 min from the return of spontaneous circulation (ROSC) in the prehospital setting.

During the study period, 158 patients were randomized of which 137 patients were included in the modified intention-to-treat-population. The primary outcome in the STEROHCA trial was IL-6 and neuron-specific enolase (NSE) as markers of systemic inflammation and neurological injury, respectively. Methylprednisolone significantly reduced IL-6 but did not affect NSE levels [5].

### Outcome

The primary outcome measured was the variation in metabolite concentrations at 48 h between the methylprednisolone and placebo groups. The secondary outcomes assessed were differences in concentrations upon hospital arrival to observe early effects. Associations of early metabolite levels and hemodynamic profile, inflammation, and markers of cerebral damage were explored.

### Metabolomics

In the current study, arterial blood samples were collected at two intervals: upon admission and 48-h post-randomization. Immediately after collection, samples were centrifuged at 2000×*g* for 10 min, and the resulting plasma was stored at −80 °C in a biobank for later analysis. The analysis was conducted by MS-Omics in Vedbæk, Denmark, using a Thermo Scientific Vanquish LC connected to an Orbitrap Exploris 240 MS from Thermo Fisher Scientific. An electrospray ionization interface served as the ionization source, and the analysis included both positive and negative ion modes with polarity switching. The UPLC analysis adhered to a modified protocol originally outlined by Doneanu et al. [10] Furthermore, gas chromatography–mass spectrometry was performed by MS-Omics as follows: samples were derivatized with methyl chloroformate using a slightly modified version of the protocol described by Smart et al. (DOI: 10.1038/nprot.2010.108). All samples were analysed in a randomized order. Analysis was performed using gas chromatography (7890B, Agilent) coupled with a quadrupole detector (5977B, Agilent. The system was controlled by ChemStation (Agilent). Raw data were converted to netCDF format using Chemstation (Agilent), before the data was imported and processed in Matlab R2021b (Mathworks, Inc.) using the PARADISe software described by Johnsen et al. (DOI: 10.1016/j.chroma.2017.04.052). Cortisol, cortisone, and methylprednisolone were measured as part of an untargeted approach with identification by accurate mass (with an accepted deviation of 3 ppm), and MS/MS spectra, and only relative quantity is known. Sixty metabolites were predetermined for quantification based on existing literature and prior research on critically ill patients [3]. All quantifications were performed using external calibration rows. Quantification of compounds analysed via the GC method was normalized to alanine-4d. Meanwhile, for those analysed via the LC method in both ionization modes, normalization involved one of five internal standards (Carnitine-d9, Glucose-13C6, Glutamic acid-d5, Phenylalanine-d8, or TMAO-d9). The selection of the appropriate internal standard was based on its effectiveness in mitigating plasma matrix effects, as determined through spiking experiments. In total, 997 out of 14,280 (6.98%) quantified measurements were below the limit of detection (LOD). 12(S)-HETE and 15(S)-HETE were below LOD in all but two samples at 48 h and were removed. The remaining were imputated with LOD × 0.5.

### Hemodynamics

To assess hemodynamic stability, we used the vasoactive-inotropic score (VIS) relative to the mean arterial blood pressure (MAP) (VIS/MAP ratio), which provides an estimate of the pharmacological circulatory support needed to obtain the targeted MAP [6] (See supplementals for details).

### Refractory post-resuscitation shock

In the modified intention-to-treat population, 13 patients died within 48 h and, as a result, did not have blood samples available at the 48-h mark. These patients constituted a specific cohort characterized by having received pharmacological circulatory support, not dying from brain herniation, and thus being defined as being in refractory post-resuscitation shock.

### Other biomarkers (IL-6 and NSE)

Biomarkers were assessed at prespecified timepoints that is at admission, and after 48 h. IL-6 levels were quantified in plasma samples treated with ethylenediamine tetraacetic acid (EDTA) using the Bio-Rad 17-plex human cytokine assay. Analyses of NSE levels were conducted utilizing a COBAS 8000 system and analyzed according to routine biochemistry protocols in a laboratory standardized to DS/EN ISO 15189.

### Statistics

Categorical data are shown as numbers or percentages and evaluated using either chi-squared or Fisher’s exact test. Continuous data are displayed as medians [25th percentile–75th percentile (IQR)] and analyzed using the Kruskal–Wallis test. Principal component analysis (PCA) was employed to identify potential outliers among the patients, with 10 patients identified as outliers after 48 h. These patients were located outside the 95% confidence ellipse (Hotelling’s T^2^ ellipse) on the first two principal components. To maintain the integrity of the primary outcome analysis (see below), these patients were not excluded. However, a sensitivity analysis was conducted by excluding these outliers to assess the robustness of the primary results.

Associations of metabolite levels at admission hours with time from ROSC to sampling, age, time to ROSC, ST-elevation in first ECG, IL-6, and creatinine were assessed in multivariable linear regression analyses (covariates specified in the text). Differences in metabolite levels between groups at hospital arrival were adjusted for possible imbalances (age, sex, time to ROSC, ST-elevation in ECG, and time from ROSC to sampling) by linear regression following log2 transformation of metabolite concentrations. Similarly, the metabolite levels at 48 h were adjusted for age, sex, time to ROSC, percutaneous coronary intervention, and if the patient required dialysis during ICU admission. The covariates were chosen as previous studies have shown them to be associated with metabolite concentrations [3, 11]. The differences between groups of log2-transformed estimated marginal means metabolite concentrations are visualized in volcano plots at both timepoints. Metabolites associated with dying within 48 h were investigated by logistic regression adjusted for randomization, ST-elevation myocardial infarct, time to ROSC, and time from ROSC to sampling.

Associations of metabolite levels at 48 h with IL-6, NSE, VIS/MAP-ratio, and 180-day mortality were assessed in multivariable linear and logistic regression analyses (covariates specified in the text) for the up and down-regulated metabolites at 48 h. In addition, the primary outcome was investigated in the subgroups of patients who remained comatose upon arrival and in patients in which the precipitated cause was myocardial infarction requiring acute percutaneous coronary intervention (PCI) or coronary artery bypass grafting.

The *p* values are reported after correction for multiple testing using the false discovery rate (FDR) method for the primary and secondary outcomes. Consequently, the widths of the confidence intervals have not been adjusted for multiplicity and should not be used as substitutes for hypothesis testing in all exploratory analyses. *p* values are two-tailed, and a *p* value below 0.05 was considered significant. Statistical analyses were performed in R (The R Foundation, v. 4.4.1).

## Results

The trial randomized 158 patients to methylprednisolone (n = 80) or placebo (n = 78), and 137 patients were included in the modified intention-to-treat population. Blood samples were available in 130 (95%) of patients, 121 (88%) at the time of hospital arrival and 117 (94% of patients alive) at 48 h; 13 patients died within 48 h (four patients in the methylprednisolone group and nine patients in the placebo group), and seven samples were missing due to logistics. The baseline characteristics of the two groups are presented in Table 1 (see Supplemental Table 1 for baseline characteristics of the patients with available blood samples at hospital arrival). Median age was 67 years (IQR: 56–75 years), and 81% were men. The median time to ROSC was 18 min (13–21 min) in the methylprednisolone group compared with 14 min (10–20 min) in the placebo group.

**Table 1:** Baseline characteristics of the modified intention-to-treat population

	Methylprednisolone, N = 68	Placebo, N = 69
Demographics		
Age in years, median (IQR)	67 (57, 74)	66 (56, 75)
Male sex, no. (%)	56 (82%)	56 (81%)
Medical History		
Hypertension, no. (%)	30 (44%)	29 (42%)
Diabetes type I or II, no. (%)	9 (13%)	6 (8.7%)
Heart failure, no. (%)	16 (24%)	12 (17%)
Cardiac arrest		
Bystander cardiopulmonary resuscitation, no. (%)	60 (88%)	56 (81%)
First monitored rhythm shockable, no. (%)	64 (94%)	65 (94%)
Time to ROSC, median (IQR)	18 (13, 20)	14 (10, 19)
ST-Elevation in first ECG, no. (%)	29 (43%)	28 (41%)
Hospital admission		
LVEF in %, median (IQR)	40 (29, 45)	40 (25, 50)
pH, median (IQR)	7.24 (7.18, 7.30)	7.24 (7.20, 7.32)
Lactate, mmol/L, median (IQR)	5.35 (3.48, 6.95)	4.50 (2.70, 6.90)
Emergency coronary angiography, no. (%)	38 (56%)	42 (61%)
Primary percutaneous coronary intervention, no. (%)	23 (34%)	25 (36%)
Time from ROSC to blood sampling, median (IQR)	2.20 (1.52, 3.54)	1.85 (1.36, 3.26)

*ROSC* return of spontaneous circulation, *LVEF* left-ventricular ejection fraction

### Early metabolic effects of methylprednisolone

Blood samples were obtained upon admission, with a median time from ROSC to sampling of 2.04 h (1.43–3.27 h). Only the short-chain acylcarnitine butyrylcarnitine (C4) was different at the time of admission [22.1% (95% CI 0.5–48.4%) higher in the placebo arm compared with methylprednisolone] (see Supplemental Fig. 1). Few trends were noted, with 48.8% (95% CI −0.4 to 122.4%) higher prostaglandin E2 levels in placebo vs. methylprednisolone, along with numerically higher levels of ω-6 fatty acids and the short chain acylcarnitine butyrylcarnitine (C4) which was 22.1% (95% CI 0.5–48.4%) higher in placebo arm compared with methylprednisolone. No differences reached statistical significance at the 0.05 level after adjustments for multiple testing. A complete list of raw median values by group can be found in Supplemental Table 2.

**Fig. 1:**
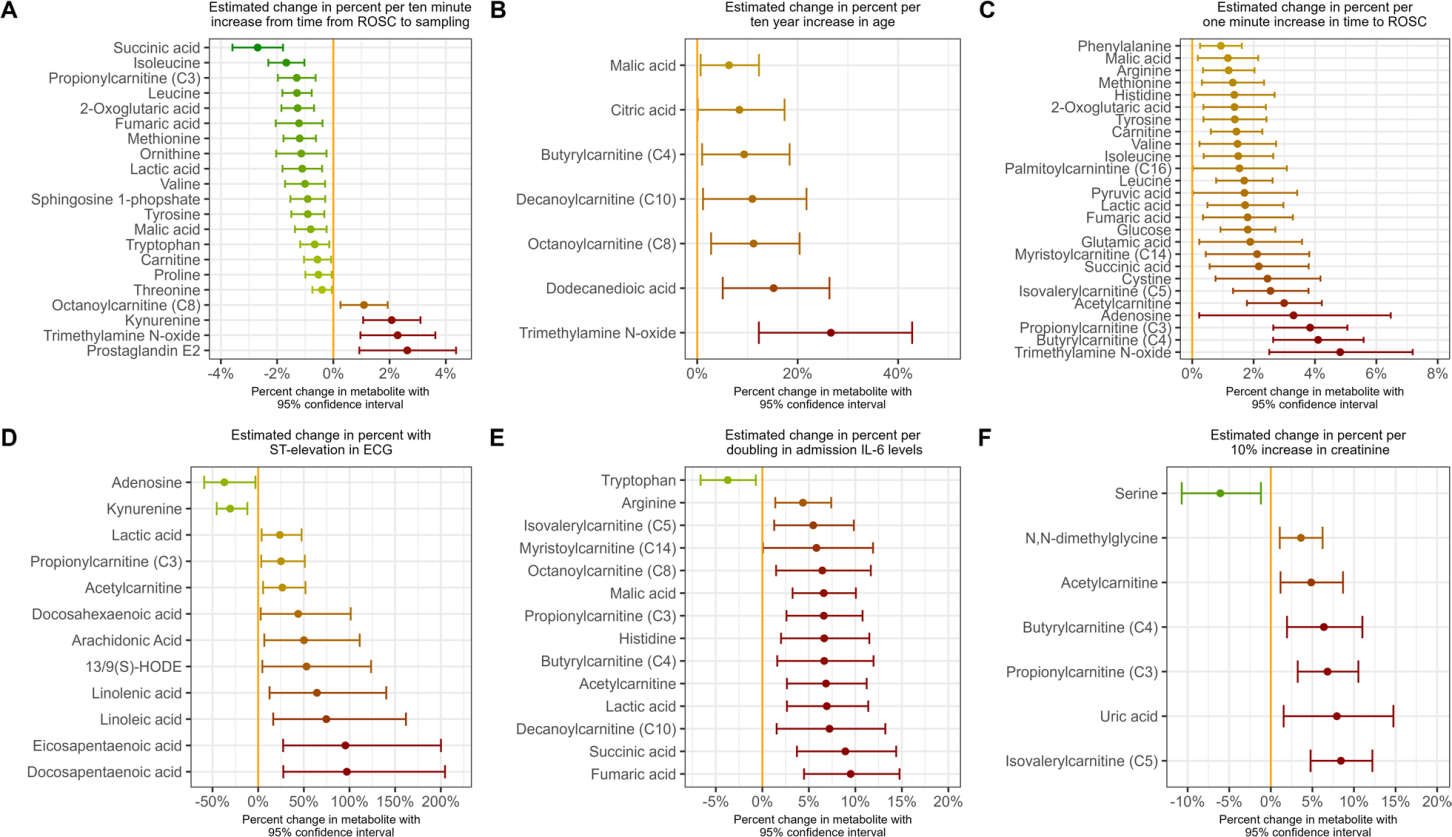
Estimates of change in percent in metabolite level at admission by differences in time from ROSC to sampling (**A**), age (**B**), time to ROSC (**C**), ST-elevation in first ECG (**D**), admission IL-6 levels (**E**), and admission creatine (**F**) with metabolite levels analyzed by multivariable linear regression. Estimates stem from different models. All models (**A–F**) were adjusted for randomization. Model **A** was adjusted for ST-elevation in the first ECG and the time to ROSC. Models **B**, **E**, and **F** were adjusted for ST-elevation in the first ECG, time from ROSC to sampling and the time to ROSC. Model **C** was adjusted for the time from ROSC to sampling and ST-elevation in first ECG. Model **D** was adjusted for the time from ROSC to sampling and time to ROSC. Only associations with a 95% confidence interval not crossing unity are shown. The confidence interval widths have not been adjusted for multiplicity and may not be used in place of hypothesis testing. *ECG* electrocardiogram, *IL-6* interleukin-6, *ROSC* return of spontaneous circulation, *CI* confidence interval

Methylprednisolone, cortisol, and cortisone were measured as part of an untargeted approach and not quantified; thus, only relative measurements are available. Methylprednisolone was 99.8% (95% CI 99.8–99.9%) lower in placebo vs. the methylprednisolone group upon admission. In contrast, cortisol and cortisone were 18.7% (95% CI 1.9–38.2%) and 148.3% (95% CI 105.8–199.5%) higher in the placebo group, respectively.

Associations of different markers with metabolites at admission were explored in multivariable regression models adjusting for possible confounders (Fig. 1 and Supplemental Fig. 2). Several metabolites from the TCA cycle (e.g., succinate acid) were associated with a longer duration of cardiac arrest, and lower levels were observed with a longer time from ROSC to sampling. Furthermore, TCA levels were associated with IL-6 levels upon admission, NSE levels at day 2, and 180-day all-cause mortality. Higher short-chain acylcarnitines levels were similarly associated with longer time to ROSC and higher IL-6 levels and creatine levels upon admission. Higher medium-chain acylcarnitines levels were associated with increasing age and IL-6 levels at admission. Higher free fatty acids (ω-6 and ω-3) levels were associated with ST elevation in the first ECG, lower levels of NSE and lower odds of 180-day all-cause mortality. Several amino acids were associated with a longer duration of cardiac arrest and similar to TCA metabolites, decreased with a longer time from ROSC to sampling. Increasing levels of tryptophan was the only metabolite associated with lower levels of IL-6 at admission. Higher kynurenine and prostaglandin E2 levels were associated with a longer time from ROSC to sampling.

**Fig. 2:**
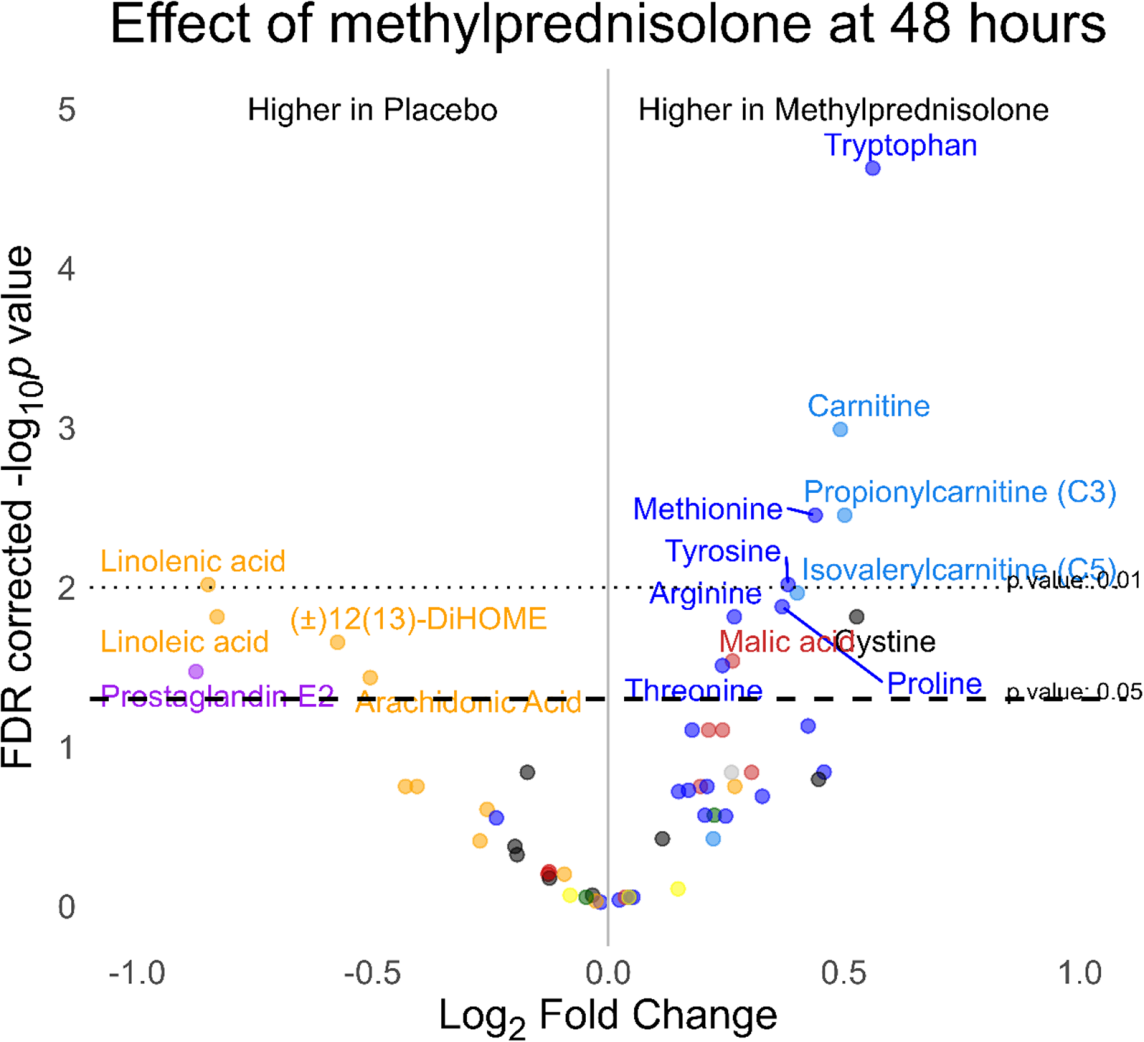
Volcano plot of metabolite concentrations at 48 h by randomization. Differences in metabolite concentrations between groups were assessed by multivariable regression adjusting for age, sex, time to return of spontaneous circulation, percutaneous coronary intervention, and need for dialysis. *p* values are adjusted for multiple testing

### Metabolic effects of methylprednisolone after 48 h

The effects of prehospital methylprednisolone 48 h after admission on plasma metabolites are shown in the volcano plot in Fig. 2. Prostaglandin E2 was 83.4% higher (95% CI 16.9–187.6%) in the placebo group compared to the methylprednisolone group, along with significantly higher levels of the free fatty acids linolenic acid, linoleic acid, (±12(13)-DiHOME, and arachidonic acid. Conversely, several amino acids, such as tryptophan (47.6% higher; 95% CI 27.9–70.2%), arginine (20.4% higher; 95% CI 6.8–35.6%), and cystine (44.1% higher; 95% CI 13.6–82.8%), as well as short-chain acylcarnitine, such as propionylcarnitine (C3) (41.6% higher; 95% CI 18.1–69.8%), were elevated in the methylprednisolone group compared to placebo (all FDR-corrected *p* values < 0.05). The kynurenine/tryptophan ratio was 60.9% (95% CI 17.7–119.9%) higher in the placebo group compared to the methylprednisolone group. A complete list of raw values by group is available in Supplemental Table 3, and temporal changes of metabolites (uncorrected for clinical imbalances between groups) per group can be seen in Supplemental Fig. 3. The results were overall consistent if patients who regained consciousness in the first hours after randomization were excluded and in patients with myocardial infarction requiring acute revascularization (results not shown). After 48 h, methylprednisolone had dropped below detection level in 46% of patients of the active group. Cortisol was 50.0% (95% CI 9.5–105.6%) higher in the placebo group, while cortisone approached unity (27.4% (95% CI −0.3 to 62.9%) higher in the placebo group). Excluding outliers did not alter the results.

**Fig. 3:**
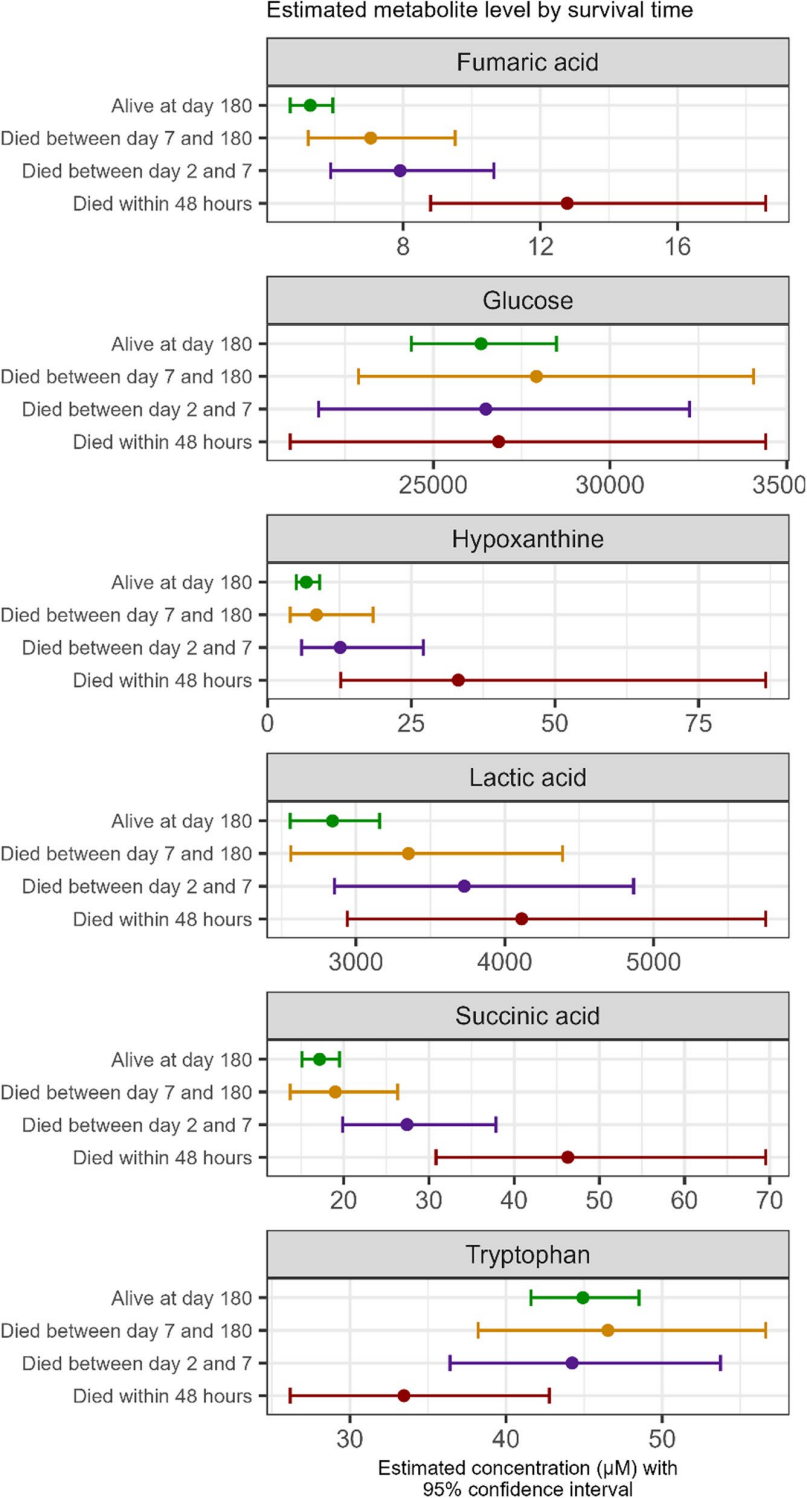
Selected metabolites at admission according to survival time (Alive at day 180: n = 86, died between day 7 and 180: n = 13, died between days 2 and 7: n = 14, died within 48 h: n = 8). Levels are reported as estimated marginal means adjusted for randomization, time to ROSC, ST-elevation in the first ECG, and time from ROSC to sampling of blood. The confidence interval widths have not been adjusted for multiplicity and may not be used in place of hypothesis testing. *ROSC* return of spontaneous circulation

### Refractory post-resuscitation shock

Thirteen patients died within 48 h. Blood samples were available at admission for metabolite quantification for eight of these patients. Median time from admission to death was 17 h (IQR: 12–23 h). These eight patients, comprising three from the methylprednisolone arm and five from the placebo arm, are categorized as being in refractory post-resuscitation shock (no one died from brain herniation). Among the various metabolites analysed, a 10% increase in admission levels of hypoxanthine [adjusted OR: 1.10 (95% CI 1.03–1.19), fumaric- [1.30 (95% CI 1.12–1.58)], and succinic acid [1.18 (95% CI 1.07–1.35)] and levels of tryptophan [0.79 (95% CI 0.61–0.99)] were associated with dying within 48 h, see Supplemental Fig. 4. Hypoxanthine, fumaric acid, and succinic acid levels were higher in patients who died sooner, whereas lower tryptophan levels were observed in patients who died from refractory post-resuscitation shock (Fig. 3).

### Inflammation, brain injury, and hemodynamic stability

Metabolite levels exhibited different associations with clinical outcomes at 48 h in both the methylprednisolone and placebo groups. A 10% increase in tryptophan was linked to a 17% (95% CI 9–24%) reduction in IL-6 levels in the placebo group, compared to a 9% (95% CI −2 to 18%) reduction in the methylprednisolone group. Prostaglandin E2 was associated with higher IL-6 levels in both groups and was linked to increased NSE levels and higher 180-day mortality in the methylprednisolone group. In addition, a 10% increase in carnitine, which was elevated in the methylprednisolone group, correlated with a 6.0% (95% CI 0.9–11.3%) increase in NSE levels. Arginine, also upregulated by methylprednisolone, was associated with worse hemodynamic status in the placebo group, indicated by a slightly higher VIS/MAP ratio. (Supplemental Fig. 5 illustrates the associations between the up-regulated metabolites and various outcomes at 48 h, while Supplemental Fig. 6 shows the same for down-regulated metabolites).

## Discussion

Patients resuscitated from OHCA face high rates of morbidity and mortality. This poor prognosis is largely attributed to ischemia–reperfusion injury, a complex metabolic disturbance with severe consequences. Therefore, an improved understanding of the metabolic disturbances and novel interventions aimed at mitigating ischemia–reperfusion injury and its aftermath are critically needed.

The primary finding of the present explorative study is that the administration of 250 mg of methylprednisolone intravenously shortly after the ROSC seems to have little immediate nongenomic effects on metabolomics but induces metabolic effects that persist for more than 48 h. Specifically, methylprednisolone: (1) decreases ω-6 fatty acids; (2) increases several amino acids, notably tryptophan; and (3) increases carnitine and short-chain acylcarnitines. While the observed metabolic differences are likely related to the administration of methylprednisolone, this study cannot determine whether these effects are directly mediated by the steroid or occur indirectly through downstream pathways, such as reduced catecholamine exposure or changes in supportive care that may follow steroid administration [6].

### Tryptophan metabolism

Higher tryptophan levels were associated with lower IL-6 levels at hospital admission and were 50% higher in patients randomized to methylprednisolone after 48 h. Tryptophan is an essential amino acid obtained solely from the diet. Besides being a building block for protein synthesis, it is metabolized through the kynurenine and serotonin pathways [12]. Studies have linked activation of the kynurenine pathway with increased mortality in resuscitated out-of-hospital cardiac arrest patients [13–15]. Consequently, it has been postulated that inhibiting the enzymes indoleamine 2,3-dioxygenase (the extrahepatically enzyme that metabolizes tryptophan to kynurenine and accounts for 99% of tryptophan metabolism during systemic inflammation [12]) could confer advantages by limiting the catalyzation of the amino acids tryptophan to kynurenine [16–18]. Indeed, recent findings demonstrated that the deletion of indoleamine 2,3-dioxygen was linked to reduced neurofilament light chain (NFL), a biomarker of brain injury, in mouse models of cardiac arrest [18]. In the present study, higher tryptophan levels were associated with lower IL-6 levels at the time of admission and in the placebo group at 48 h. Higher tryptophan levels were also associated with lower NSE levels in the methylprednisolone group.

The glucocorticoid dexamethasone has been reported to enhance the induction of indoleamine 2,3-dioxygenase by the pro-inflammatory cytokine interferon-γ [12]. Still, we found higher tryptophan plasma levels, alongside a lower kynurenine/tryptophan ratio suggesting a lower activity of indoleamine 2,3-dioxygen with 250 mg pulse-dose methylprednisolone. Our results are in line with a study in healthy young males treated with 4 mg dexamethasone (approximately equivalent to 20 mg of methylprednisolone), showing increased tryptophan levels after 24 h, and a study of patients with COVID-19 treated with methylprednisolone [19, 20].

Furthermore, targeted temperature management to 33 °C resulted in lower tryptophan levels compared with 36 °C h after normothermia had been reached in a sub-study of the TTM trial [21, 22]. In a recent study, our research group demonstrated that inhibiting the IL-6 receptor with tocilizumab significantly increased plasma levels of several amino acids compared to placebo in a similar cohort of patients. Notably, however, plasma tryptophan levels were similar 48 h after tocilizumab compared to placebo [11]. The overall higher levels of plasma amino acids effect are potentially attributed to a reduction in acute phase protein synthesis, thus a smaller requirement for amino acids. Indeed, methylprednisolone resulted in lower levels of C-reactive protein [5].

### Inhibition of prostaglandin E2

Methylprednisolone decreased the levels of both arachidonic acid as well as prostaglandin E2. This effect likely reflects methylprednisolone's well-known downregulation of phospholipase A2 [23]. Phospholipase A2 hydrolyses phospholipid in the cell membrane and hereby causes a release of arachidonic acid, which can be subsequently metabolized to prostaglandins and thromboxane by cyclooxygenase (COX). Prostaglandin E2 plays a role in all the classic signs of inflammation, including redness and edema, by increased blood flow due to arterial dilation and heightened microvascular permeability. It also crosses the blood–brain barrier and regulates the febrile response [24, 25]. COX inhibition is a key mechanism by which aspirin exerts its therapeutic effect in the management of myocardial infarction by decreasing thromboxane. Interestingly, the addition of glucocorticoids to aspirin has been reported to reduce thromboxane levels compared to aspirin alone [26]. This add-on effect could be involved in the reduced troponin levels observed in patients with ST-elevation myocardial infarction randomized two prehospital pulse-dose methylprednisolone in the PULSE-MI trial [27].

### Free fatty metabolism and mitochondrial function

Carnitine and short-chain acylcarnitines were higher in patients treated with methylprednisolone and further associated with a worse prognosis in this group. Higher levels of carnitine, along with elevated free fatty acids, may suggest a metabolic state characterized by reduced beta-oxidation in the mitochondria. Carnitine is essential for transporting fatty acids into mitochondria for oxidation. However, when beta-oxidation is impaired or less active, free fatty acids may accumulate in the bloodstream, and carnitine levels rise due to decreased utilization. The mitochondrial effects induced by glucocorticoids depend on the treatment dose and duration of exposure: Acute exposure has been shown to enhance the mitochondria's capacity to produce energy [28]. However, high doses of glucocorticoids have been found to result in decreased levels of ATP in the brain, as reviewed by Jaszczyk et al. [29]. The link between higher acylcarnitines and NSE levels as a marker of brain injury observed in the present study is likely a confounder as beta-oxidation is not favoured in the brain, especially in the neurons [30].

Overall, the results suggest potential side effects of methylprednisolone and underscore the uncertainty surrounding the clinical impact of glucocorticoid treatment [31]. However, levels of free fatty acids should be interpreted cautiously in OHCA cohorts, as this study, in line with previous findings, found a strong association between heparin administration (in STEMI patients) and increased levels [3].

### Adrenal insufficiency and hemodynamic stability

An inadequate adrenal response has been associated with early refractory shock after resuscitation and glucocorticoids might be beneficial by mitigating vasoplagia and increasing affinity for catecholamines [32]. Exogenous glucocorticoid administration reduces endogenous adrenal steroids' production through a negative feedback mechanism [19, 33]. Consistent with this, we observed significantly lower levels of both cortisol and cortisone already 2 h after administration of methylprednisolone compared with placebo, differences that persisted for at least 48 h. This indicates that methylprednisolone provided very early effects that may be beneficial in securing hemodynamic stability [7]. The STEROHCA trial did not find a signal in reducing the extent of brain injury assessed by the marker NSE, still, a lower adjusted hazard for all-cause mortality was found in patients treated with methylprednisolone. A substudy of the present trial showed generally enhanced hemodynamic in the group receiving glucocorticoids [6]. Lower levels of arginine, which were higher in methylprednisolone-treated patients, have been associated with cardiogenic shock, as arginine is the precursor of nitric oxide synthesis [34]. However, we also found a reverse correlation in the placebo group with the VIS/MAP ratio, indicating a slightly worse hemodynamic profile with increasing arginine. Whether the positive effect observed could be due to early stabilizing effects on the circulation remains unknown.

### Post-resuscitation shock

Identifying patients who are in, remain in, or may deteriorate into shock due to the initial cause or ischemic reperfusion injury is crucial for reducing mortality after OHCA. Timely intervention with mechanical circulatory support or steroid treatment could be beneficial in improving outcomes for these patients. A previous study retrospectively identified longer low-flow times and higher lactate levels as predictors of a higher hazard of refractory post-resuscitation shock [35]. Similarly, the CREST model predicts cardiovascular etiology death in non-STEMI OHCA patients including known history of coronary artery disease and non-shockable rhythm [36]. The cause of death varies according to survival time, with many patients succumbing within the first 48 h primarily due to refractory post-resuscitation shock [35]. The STEROHCA trial included patients in the pre-hospital setting, unlike many other OHCA trials that enroll patients only after hospital admission. This approach enabled us to study a more heterogeneous real-world population of patients, including those in more severe conditions, such as refractory post-resuscitation shock, upon hospital arrival. Consequently, it allowed the inclusion of patients who would have been excluded from other trials [22, 37].

In the present study, metabolites such as succinic acid, hypoxanthine, and tryptophan appeared to more effectively distinguish patients who succumbed to refractory post-resuscitation shock compared to lactic acid and glucose [38]. Metabolic acidosis (e.g., higher succinic acid and lactic acid), is a known marker associated with mortality in OHCA patients [39]. Understanding the aetiology of this acidosis provides more precise insights for addressing the underlying causes. Hypoxanthine, an intermediate in the purine degradation pathway, is broken down into xanthine and uric acid by xanthine oxidase. During hypoxia, the degradation of adenine nucleotides to hypoxanthine accelerates, but its conversion to xanthine and uric acid slows due to the oxygen dependency of xanthine oxidase. Elevated hypoxanthine levels serve as potential markers of hypoxia in various diseases [40]. Future studies should evaluate whether it better discriminates between OHCA patients who are to recover swiftly hemodynamically and OHCA patients who remain unstable in whom routine treatment escalation could be lifesaving.

### Limitations

The intervention was administered in the pre-hospital setting to enable early modulation of pathophysiological processes. As a result, no baseline blood samples were obtained prior to treatment, and we cannot exclude the possibility that pre-existing metabolic differences between groups may have influenced our findings. The present sub-study was limited to patients with available plasma samples, comprising 130 of 137 patients in the modified intention-to-treat cohort (95%). While this high capture rate supports internal validity, we acknowledge that the analysis is no longer a complete cohort analysis, and unmeasured confounders due to missing data may have influenced the results. Although the study was originally randomized, caution is warranted in interpreting causality, and findings should be viewed in this context.

We evaluated changes in targeted metabolites at 48 h; given that the difference in IL-6 levels was highest at 24 h in the main trial, other metabolite effects may have been observed 24 h from admission. Still, both a higher ratio of methylprednisolone and a lower ratio of cortisol were observed at 48 h, proving that the effects of the drug were indeed still present at 48 h. The associations of log-transformed metabolites with clinical variables were investigated by linear regression; however, other non-linear associations may have been missed. The concentrations of metabolites have not been externally validated, and the absolute values should be interpreted with caution. The number of patients dying from refractory post-resuscitation shock was small because of our selection criteria and a good prehospital system in a mainly urban part of Denmark. The study only included patients resuscitated from an OHCA of presumed cardiac cause and with less than 30 min to ROSC and primarily men and though the cohort is similar to other and larger OHCA studies, caution should be taken if trying to extrapolate to other cohorts. Except for the metabolic effects of methylprednisolone at hospital arrival and after 48 h, outcomes were not corrected for multiplicity, nor were they pre-specified, and due to the high risk of type I error, the confidence intervals should not be used for testing. In addition, no power calculation was conducted for the metabolites investigated, and the study might have been underpowered to detect additional differences. Collectively, the study should be considered hypothesis-generating, and findings interpreted in the context of its exploratory design.

## Conclusion

In this exploratory study, early administration of 250 mg of methylprednisolone after resuscitation appeared to drive sustained metabolic effects over 48 h. Specifically, methylprednisolone led to reductions in ω-6 fatty acids and increases in several amino acids, with a notable rise in tryptophan.

## Supplementary Information

Additional file 1.

## Data Availability

Data are available upon reasonable request to the authors.
