# Supplementary material for: Metabolic effects of high-dose glucocorticoid following out-of-hospital cardiac arrest
Source: Intensive Care Med Exp. 2025 Apr 26;13:46. doi: 10.1186/s40635-025-00754-8 (PMC12033126; doi:10.1186/s40635-025-00754-8)
Supplement: Supplementary file 1 — Additional file 1. [file 40635_2025_754_MOESM1_ESM.docx]

Supplementals

Metabolic Effects of Methylprednisolone Treatment in Out-of-Hospital Cardiac Arrest Patients

Sub-Study of the STEROHCA Trial

Beske et al.

### Vasoactive inotropic score and MAP

Vasoactive inotropic score: Dopamine (µg/kg/min) + dobutamine (µg/kg/min) + 100 x adrenaline (µg/kg/min) + 100 x noradrenaline (µg/kg/min) + 10 x milrinone (µg/kg/min) + 50 x levosimendan (µg/kg/min) + 10000 x vasopressin (U/kg/min)

Mean arterial blood pressure (MAP) was calculated based on the systolic and diastolic blood pressure measured by an arterial catheter by the formula:

MAP = 1/3 x (systolic blood pressure) + 2/3 x (diasystolic blood pressure)

The values were electronically stored

**Supplemental Figure 1**


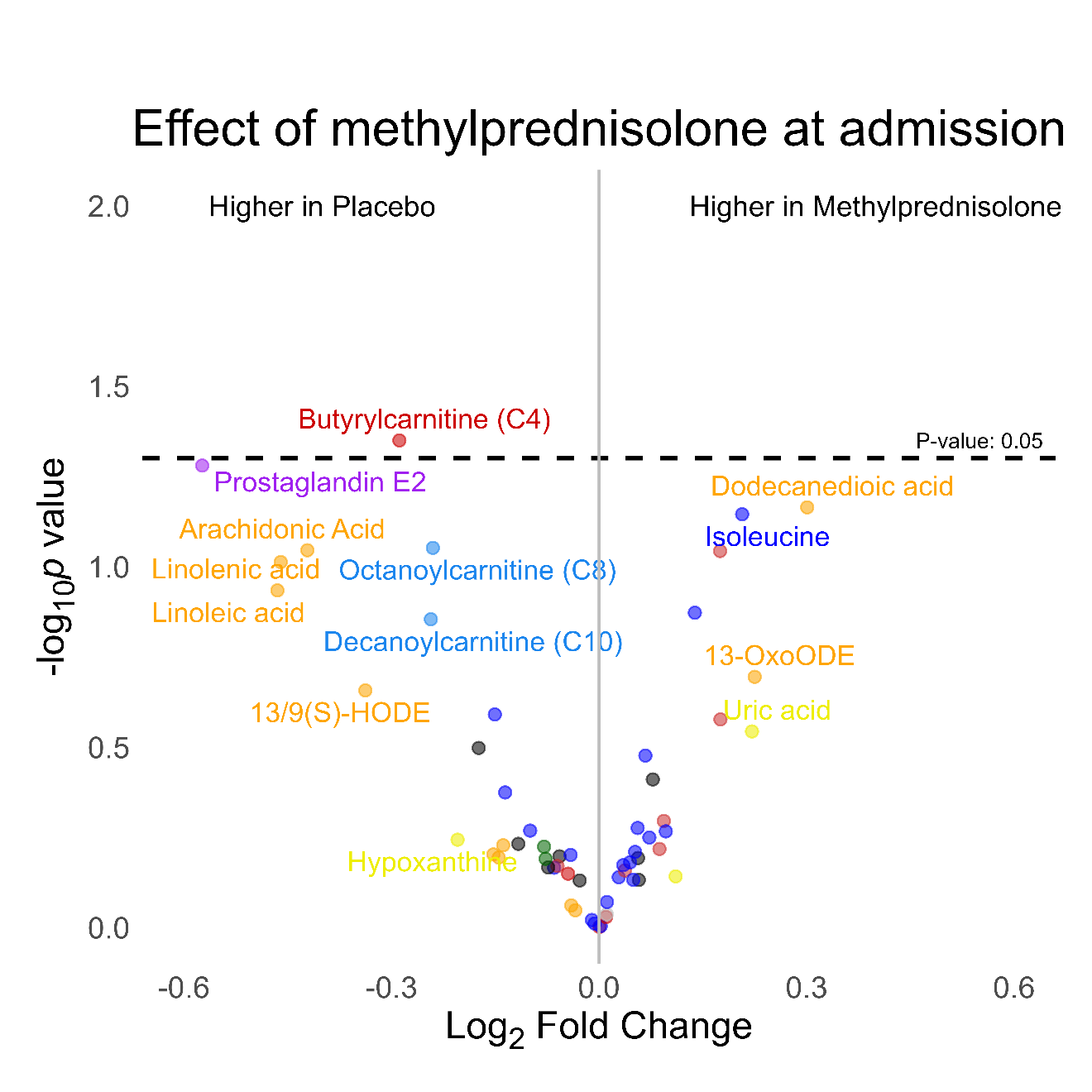


Volcano plot of metabolite concentrations at admission by randomization.

Differences in metabolite concentrations between groups were assessed by multivariable regression adjusting for age, sex, time to return of spontaneous circulation, percutaneous coronary intervention, and need for dialysis. P-values are unadjusted for multiple comparisons. No metabolites reached statistical significance after adjustments for multiple testing.

**Supplemental Figure 2**


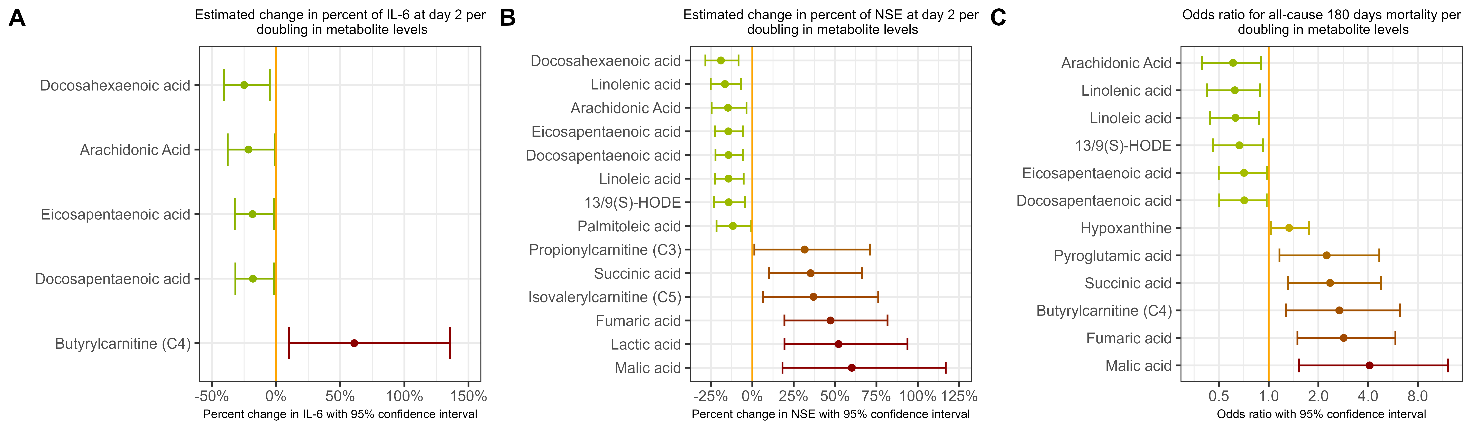


Estimates of change in IL-6 levels at day 2 (**A**), NSE levels at day 2 (**B**), and odds of dying within 180 days (**C**) by *metabolite levels at admission*. Associations were analyzed by linear or logistic multivariable regression. All models (**A-C**) were adjusted for randomization, ST-elevation in the first ECG, time to ROSC, and time from ROSC to sampling. Only associations with a 95% confidence interval not crossing unity are shown. The confidence interval widths have not been adjusted for multiplicity and may not be used in place of hypothesis testing. Abbreviations: ECG: Electrocardiogram, IL-6: Interleukin-6, ROSC: Return of spontaneous circulation, NSE: Neuron Specific Enolase, CI: Confidence interval.

**Supplemental Figure 3**


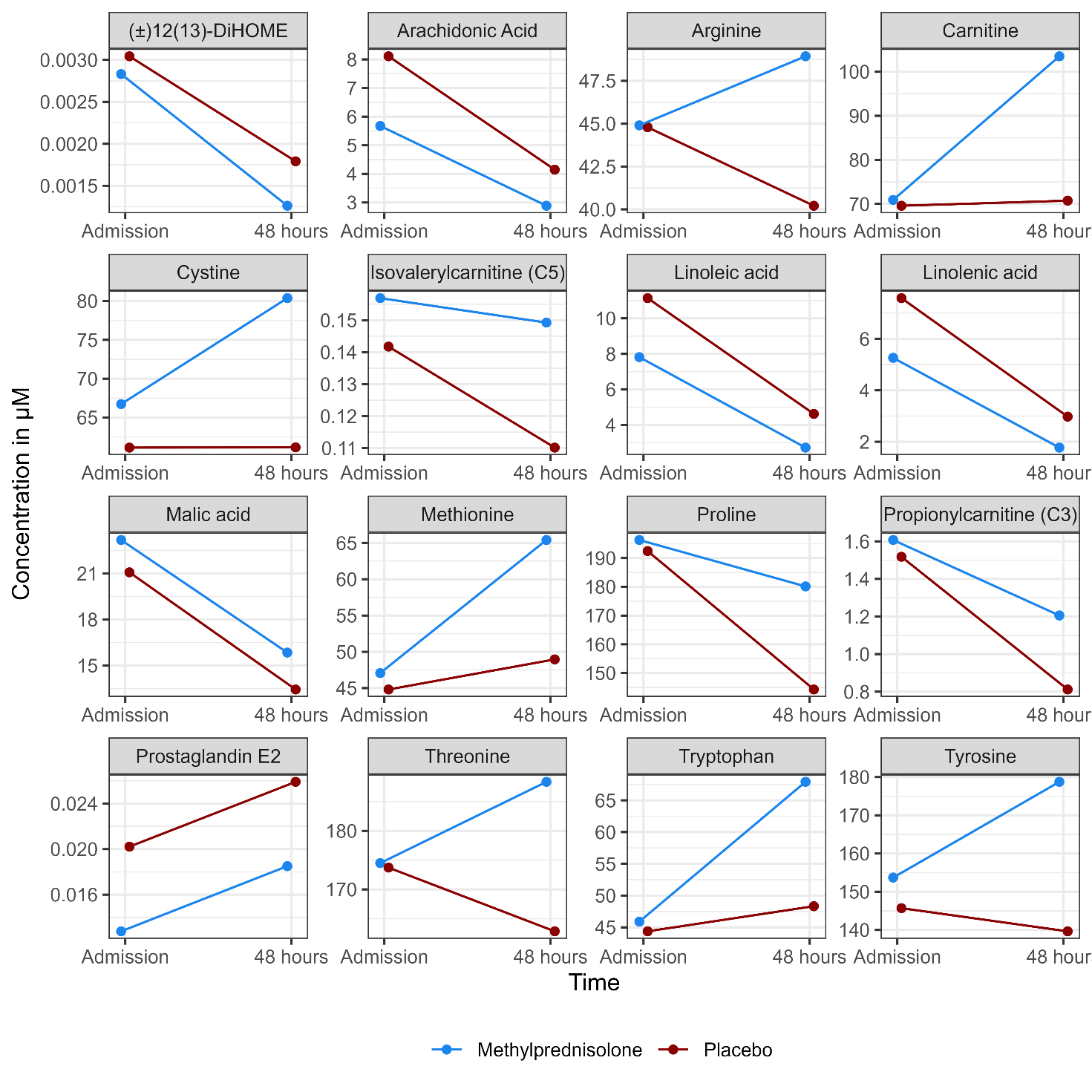
 Temporal differences in metabolite concentrations (µM) between groups. Only metabolites that differed significantly between methylprednisolone and placebo at 48 hours after correction for multiple testing are shown. Results are from a linear mixed model including time, randomization, and the randomization-by-time interactions as fixed effects and with an unstructured covariance pattern to account for repeated measurements on each patient. All metabolites were log-transformed before analyses. The results are visualized as group-specific “medians” after back-transformation. Missing data were handled implicitly via maximum likelihood estimation in the linear mixed models.

**Supplemental Figure 4**


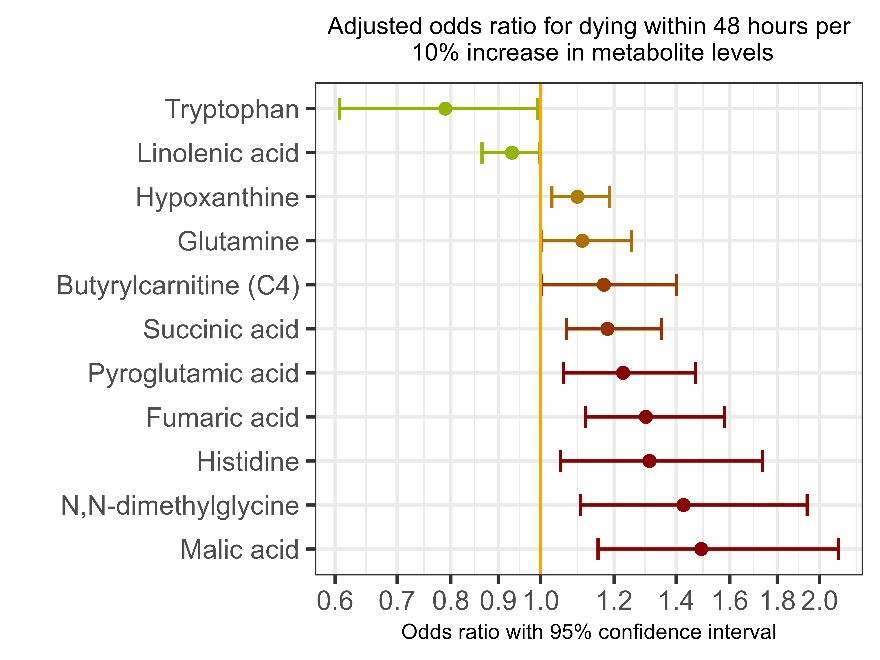


Adjusted odds ratios of dying within 48 hours (n=8) by a 10% increase in metabolite level at admission. Adjusted for time to ROSC, time from ROSC to sampling, randomization, and ST-elevation in the first ECG. Only associations with a 95% confidence interval not crossing unity are shown. The confidence interval widths have not been adjusted for multiplicity and may not be used in place of hypothesis testing.

**Supplemental Figure 5** Metabolites up-regulated by methylprednisolone


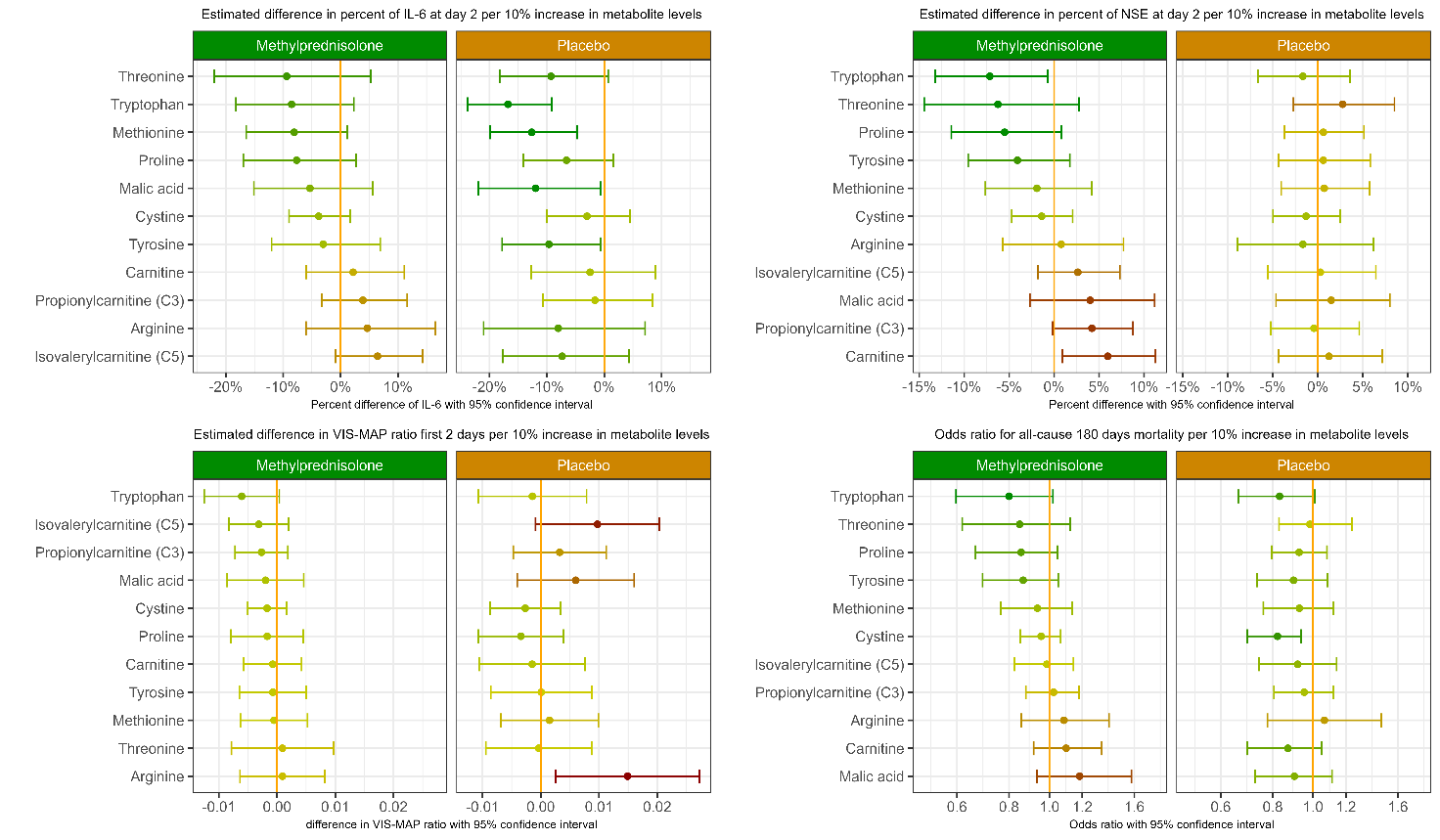


**Supplemental Figure 6** Metabolites down-regulated by methylprednisolone


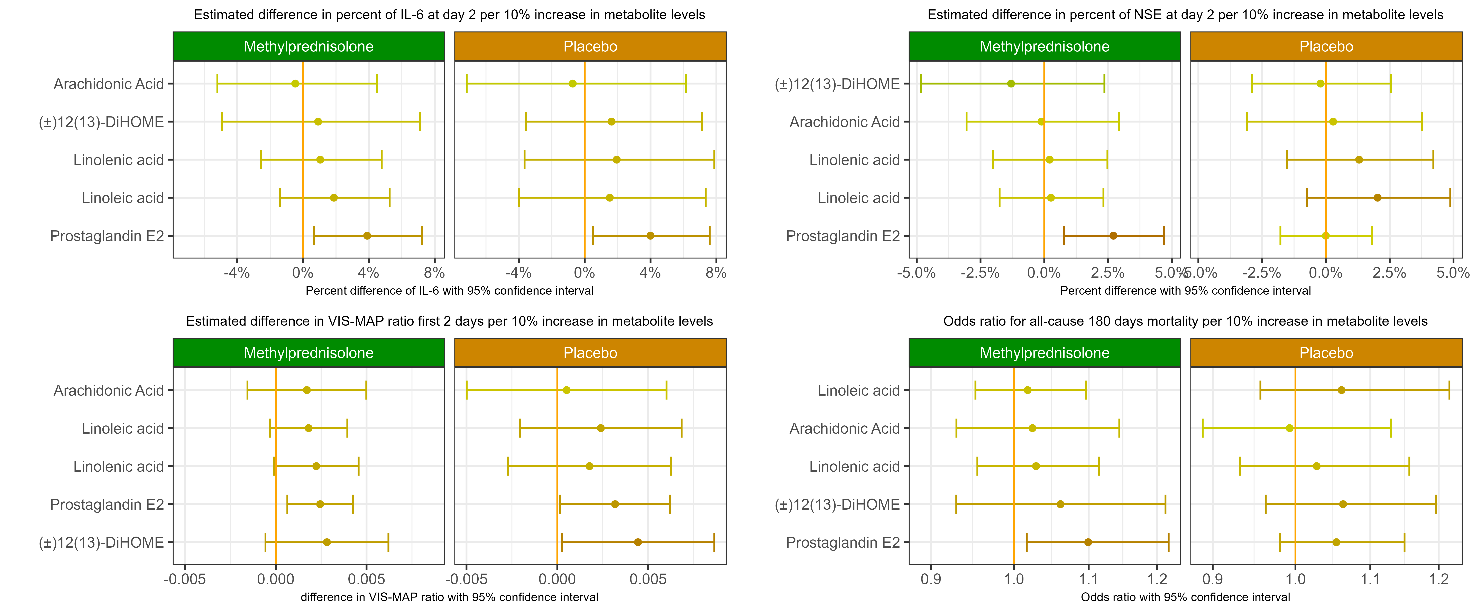


**Figure 5 and 6:** Figure 5: Associations of metabolites upregulated by methylprednisolone at 48 hours with IL-6 and NSE at 48 hours, the average VIS/MAP ratio until 48 hours, and 180 days mortality.

Figure 6: Associations of metabolites down-regulated by methylprednisolone at 48 hours with IL-6 and NSE at 48 hours, the average VIS/MAP ratio until 48 hours, and 180 days mortality. For both figures: The confidence interval widths have not been adjusted for multiplicity and may not be used in place of hypothesis testing. Abbreviations: IL-6: Interleukin 6, NSE: neuron-specific enolase, VIS / MAP ratio: Vasoactive inotropic score / Mean arterial blood pressure ratio.

**Supplemental Table 1**

| Baseline characteristics of patients with available blood samples at admission | | |
| --- | --- | --- |
|  | **Methylprednisolone**, N = 63 | **Placebo**, N = 59 |
| **Demographics** |  |  |
| Age in years, median (IQR) | 67 (56, 72) | 66 (56, 75) |
| Male sex, no. (%) | 52 (83%) | 49 (83%) |
| **Medical history** |  |  |
| Hypertension, no. (%) | 26 (41%) | 24 (41%) |
| Diabetes type I or II, no. (%) | 8 (13%) | 4 (6.8%) |
| Heart Failure, no. (%) | 14 (22%) | 7 (12%) |
| Bystander cardiopulmonary resuscitation, no. (%) | 56 (89%) | 49 (83%) |
| First monitored rhythm shockable, no. (%) | 59 (94%) | 57 (97%) |
| Time to ROSC, median (IQR) | 18 (13, 20) | 14 (9, 19) |
| ST-Elevation in first ECG, no. (%) | 29 (46%) | 27 (46%) |
| **Hospital admission** |  |  |
| LVEF in %, median (IQR) | 40 (30, 48) | 40 (25, 50) |
| pH, median (IQR) | 7.24 (7.18, 7.30) | 7.27 (7.22, 7.32) |
| Lactate, mmol/L, median (IQR) | 5.35 (3.78, 6.80) | 4.40 (2.70, 6.45) |
| Emergency coronary angiography, no. (%) | 38 (60%) | 41 (69%) |
| Primary percutaneous coronary intervention, no. (%) | 23 (37%) | 25 (42%) |
|  |  |  |
| Time from ROSC to blood sampling, median (IQR) | 2.20 (1.52, 3.54) | 1.85 (1.36, 3.26) |

**Supplemental Table 2**

| Median (IQR) metabolite concentration at hospital admission | | |
| --- | --- | --- |
| **Metabolites** | **Methylprednisolone**, N = 63 | **Placebo**, N = 59 |
| (±)12(13)-DiHOME, µM | 0.003 (0.001, 0.006) | 0.003 (0.002, 0.006) |
| 12(S)-HETE, µM | 0.0016 (0.0016, 0.0016) | 0.0016 (0.0016, 0.0016) |
| 13/9(S)-HODE, µM | 0.010 (0.005, 0.020) | 0.011 (0.007, 0.020) |
| 13-OxoODE, µM | 0.010 (0.007, 0.011) | 0.009 (0.007, 0.011) |
| 15(S)-HETE, µM | 0.0010 (0.0010, 0.0010) | 0.0010 (0.0010, 0.0010) |
| 2-Oxoglutaric acid, µM | 30 (24, 45) | 26 (21, 32) |
| Acetylcarnitine, µM | 12 (9, 18) | 12 (9, 17) |
| Adenosine, µM | 0.01 (0.01, 0.03) | 0.01 (0.01, 0.01) |
| Alanine, µM | 373 (241, 535) | 349 (279, 469) |
| Arachidonic Acid, µM | 5 (3, 11) | 7 (4, 17) |
| Arginine, µM | 46 (36, 57) | 45 (38, 55) |
| Aspartic acid, µM | 44 (35, 66) | 44 (36, 60) |
| Butyrylcarnitine (C4), µM | 0.55 (0.37, 0.85) | 0.52 (0.38, 0.78) |
| Carnitine, µM | 70 (61, 94) | 67 (59, 89) |
| Citric acid, µM | 602 (442, 909) | 643 (520, 873) |
| Cystine, µM | 82 (50, 97) | 64 (47, 94) |
| Decanoylcarnitine (C10), µM | 0.34 (0.22, 0.51) | 0.42 (0.29, 0.57) |
| Dihomo-gamma-Linolenic acid, µM | 2.32 (1.79, 3.29) | 2.54 (1.76, 4.02) |
| Docosahexaenoic acid, µM | 5 (3, 11) | 6 (3, 8) |
| Docosapentaenoic acid, µM | 3 (1, 9) | 4 (2, 8) |
| Dodecanedioic acid, µM | 0.10 (0.07, 0.16) | 0.10 (0.07, 0.12) |
| Eicosapentaenoic acid, µM | 1.09 (0.50, 3.01) | 1.22 (0.64, 2.82) |
| Fumaric acid, µM | 5.5 (4.2, 9.0) | 5.4 (3.9, 7.7) |
| Glucose, µM | 29,399 (22,126, 36,048) | 26,478 (18,241, 31,590) |
| Glutamic acid, µM | 238 (139, 321) | 217 (156, 335) |
| Glutamine, µM | 82 (51, 113) | 66 (44, 101) |
| Glycine, µM | 202 (174, 235) | 201 (180, 218) |
| Histidine, µM | 40 (29, 52) | 40 (32, 50) |
| Hypoxanthine, µM | 8 (3, 18) | 11 (2, 21) |
| Isoleucine, µM | 115 (75, 159) | 85 (67, 115) |
| Isovalerylcarnitine (C5), µM | 0.15 (0.11, 0.22) | 0.14 (0.11, 0.18) |
| Kynurenine, µM | 0.61 (0.36, 1.13) | 0.74 (0.45, 1.20) |
| Lactic acid, µM | 2,888 (2,044, 4,517) | 2,877 (1,880, 4,262) |
| Leucine, µM | 203 (149, 279) | 167 (138, 207) |
| Linoleic acid, µM | 10 (5, 15) | 12 (7, 21) |
| Linolenic acid, µM | 7 (3, 10) | 8 (4, 14) |
| Lysine, µM | 451 (285, 694) | 427 (318, 564) |
| Malic acid, µM | 24 (18, 32) | 23 (17, 29) |
| Methionine, µM | 49 (37, 64) | 45 (35, 56) |
| Myristoylcarnitine (C14), µM | 0.15 (0.11, 0.22) | 0.14 (0.11, 0.20) |
| N,N-dimethylglycine, µM | 28 (23, 32) | 26 (22, 33) |
| Octanoylcarnitine (C8), µM | 0.19 (0.14, 0.26) | 0.23 (0.16, 0.31) |
| Ornithine, µM | 136 (105, 188) | 123 (97, 162) |
| Palmitoleic acid, µM | 19 (10, 27) | 20 (9, 32) |
| Palmitoylcarnintine (C16), µM | 0.35 (0.27, 0.55) | 0.38 (0.32, 0.50) |
| Phenylalanine, µM | 92 (77, 111) | 81 (75, 101) |
| Proline, µM | 206 (171, 237) | 201 (175, 224) |
| Propionylcarnitine (C3), µM | 1.89 (1.09, 2.33) | 1.51 (1.10, 2.50) |
| Prostaglandin E2, nmol | 11 (3, 29) | 20 (10, 42) |
| Pyroglutamic acid, µM | 2.83 (2.26, 4.45) | 2.82 (2.11, 3.88) |
| Pyruvic acid, µM | 342 (219, 452) | 262 (201, 438) |
| Serine, µM | 138 (90, 180) | 125 (89, 181) |
| Sphingosine 1-phopshate, µM | 1.32 (1.01, 1.61) | 1.40 (1.06, 1.67) |
| Succinic acid, µM | 18 (13, 31) | 16 (12, 29) |
| Threonine, µM | 181 (158, 200) | 177 (164, 192) |
| Trimethylamine N-oxide, µM | 13 (8, 21) | 11 (6, 18) |
| Tryptophan, µM | 46 (36, 58) | 42 (34, 52) |
| Tyrosine, µM | 160 (117, 203) | 140 (118, 188) |
| Uric acid, µM | 84 (56, 117) | 74 (54, 117) |
| Valine, µM | 284 (205, 383) | 251 (196, 325) |

**Supplemental Table 3**

| Median (IQR) metabolite concentration at 48 hours | | |
| --- | --- | --- |
| **Metabolites** | Methylprednisolon, N = 59 | Placebo, N = 58 |
| (±)12(13)-DiHOME, µM | 0.0007 (0.0007, 0.0022) | 0.0019 (0.0007, 0.0032) |
| 13/9(S)-HODE, µM | 0.003 (0.001, 0.006) | 0.004 (0.003, 0.006) |
| 13-OxoODE, µM | 0.008 (0.006, 0.011) | 0.007 (0.006, 0.009) |
| 2-Oxoglutaric acid, µM | 32 (21, 39) | 27 (21, 35) |
| Acetylcarnitine, µM | 6 (5, 10) | 7 (5, 13) |
| Adenosine, µM | 0.008 (0.008, 0.008) | 0.008 (0.008, 0.008) |
| Alanine, µM | 332 (203, 506) | 255 (181, 391) |
| Arachidonic Acid, µM | 3.36 (2.22, 4.75) | 4.77 (3.53, 5.60) |
| Arginine, µM | 51 (39, 63) | 41 (34, 47) |
| Aspartic acid, µM | 42 (32, 60) | 42 (32, 63) |
| Butyrylcarnitine (C4), µM | 0.61 (0.35, 1.04) | 0.55 (0.32, 0.70) |
| Carnitine, µM | 96 (74, 138) | 73 (60, 88) |
| Citric acid, µM | 386 (271, 569) | 368 (281, 496) |
| Cystine, µM | 79 (62, 112) | 76 (45, 94) |
| Decanoylcarnitine (C10), µM | 0.33 (0.24, 0.52) | 0.34 (0.23, 0.55) |
| Dihomo-gamma-Linolenic acid, µM | 2.37 (1.76, 3.19) | 2.79 (1.90, 4.60) |
| Docosahexaenoic acid, µM | 2.63 (1.83, 3.66) | 2.95 (2.25, 4.20) |
| Docosapentaenoic acid, µM | 1.34 (0.93, 1.88) | 1.72 (1.18, 2.39) |
| Dodecanedioic acid, µM | 0.08 (0.05, 0.09) | 0.06 (0.05, 0.11) |
| Eicosapentaenoic acid, µM | 0.46 (0.32, 0.64) | 0.59 (0.41, 0.81) |
| Fumaric acid, µM | 3.95 (3.06, 4.88) | 3.30 (2.61, 4.18) |
| Glucose, µM | 20,855 (15,810, 26,858) | 21,881 (17,143, 25,634) |
| Glutamic acid, µM | 186 (106, 297) | 178 (112, 287) |
| Glutamine, µM | 77 (48, 144) | 74 (47, 124) |
| Glycine, µM | 209 (175, 234) | 196 (170, 227) |
| Histidine, µM | 36 (28, 44) | 32 (24, 42) |
| Hypoxanthine, µM | 14 (10, 19) | 16 (8, 21) |
| Isoleucine, µM | 120 (75, 148) | 97 (71, 133) |
| Isovalerylcarnitine (C5), µM | 0.15 (0.10, 0.22) | 0.11 (0.09, 0.13) |
| Kynurenine, µM | 0.50 (0.38, 0.84) | 0.50 (0.31, 0.86) |
| Lactic acid, µM | 1,994 (1,609, 2,342) | 1,686 (1,347, 2,214) |
| Leucine, µM | 196 (142, 232) | 165 (126, 209) |
| Linoleic acid, µM | 4.0 (1.5, 5.8) | 5.3 (2.8, 7.3) |
| Linolenic acid, µM | 1.78 (0.99, 3.73) | 3.01 (1.90, 4.82) |
| Lysine, µM | 507 (324, 702) | 412 (247, 582) |
| Malic acid, µM | 16.0 (12.7, 19.3) | 13.6 (11.1, 17.0) |
| Methionine, µM | 73 (46, 87) | 49 (37, 70) |
| Myristoylcarnitine (C14), µM | 0.13 (0.08, 0.20) | 0.11 (0.08, 0.14) |
| N,N-dimethylglycine, µM | 18 (15, 22) | 20 (17, 25) |
| Octanoylcarnitine (C8), µM | 0.18 (0.12, 0.26) | 0.18 (0.13, 0.28) |
| Ornithine, µM | 144 (103, 178) | 130 (85, 160) |
| Palmitoleic acid, µM | 7 (4, 14) | 7 (5, 15) |
| Palmitoylcarnintine (C16), µM | 0.35 (0.21, 0.53) | 0.33 (0.21, 0.45) |
| Phenylalanine, µM | 118 (92, 147) | 106 (91, 127) |
| Proline, µM | 193 (148, 231) | 158 (126, 194) |
| Propionylcarnitine (C3), µM | 1.19 (0.79, 1.63) | 0.79 (0.63, 1.07) |
| Prostaglandin E2, nmol | 15 (8, 40) | 34 (11, 62) |
| Pyroglutamic acid, µM | 2.22 (1.64, 3.24) | 2.46 (1.83, 4.01) |
| Pyruvic acid, µM | 264 (164, 425) | 245 (147, 301) |
| Serine, µM | 147 (92, 203) | 116 (88, 184) |
| Sphingosine 1-phopshate, µM | 1.15 (0.93, 1.60) | 1.18 (0.86, 1.47) |
| Succinic acid, µM | 9.23 (7.73, 11.80) | 8.15 (7.01, 9.88) |
| Threonine, µM | 199 (162, 226) | 173 (154, 194) |
| Trimethylamine N-oxide, µM | 6 (4, 14) | 5 (3, 9) |
| Tryptophan, µM | 74 (54, 88) | 50 (35, 63) |
| Tyrosine, µM | 181 (136, 235) | 140 (112, 201) |
| Uric acid, µM | 57 (34, 84) | 48 (33, 70) |
| Valine, µM | 269 (173, 366) | 219 (171, 351) |
